# Enhanced Myeloid Leukocytes in Obese Children and Adolescents at Risk for Metabolic Impairment

**DOI:** 10.3389/fendo.2020.00327

**Published:** 2020-05-27

**Authors:** Cecilia Gállego-Suárez, Ayse Bulan, Emily Hirschfeld, Phillip Wachowiak, Simin Abrishami, Cameron Griffin, Julie Sturza, Abigail Tzau, Taryn Hayes, Susan J. Woolford, Carey N. Lumeng, Joyce M. Lee, Kanakadurga Singer

**Affiliations:** ^1^Division of Pediatric Endocrinology, Department of Pediatrics, University of Michigan, Ann Arbor, MI, United States; ^2^Department of Pediatrics, Child Health Evaluation and Research Center (CHEAR), University of Michigan, Ann Arbor, MI, United States; ^3^Woodson Biostatistics Consultation Program, Department of Pediatrics, University of Michigan, Ann Arbor, MI, United States; ^4^Graduate Program in Immunology, University of Michigan Medical School, Ann Arbor, MI, United States; ^5^Graduate Program in Cellular and Molecular Biology, University of Michigan Medical School, Ann Arbor, MI, United States

**Keywords:** pediatric obesity, inflammation, insulin resistance, monocytes, CRP

## Abstract

**Objective:** We aimed to examine if myeloid leukocyte profiles are associated with metabolic impairment in children and adolescents with obesity, and if sex, age, or race influence this relationship.

**Methods:** 282 children ages 8–17 were evaluated. Predictor measures were absolute neutrophil counts (ANC), absolute monocyte count, monocyte subtypes and C reactive protein (CRP). Outcome variables were waist circumference, fasting glucose and insulin, HOMA-IR, HbA1c (%) and lipid profiles. Pearson correlation coefficients were used to determine associations between predictor and outcome variables. Wilcoxon two-sample tests were used to evaluate differences by sex.

**Results:** CRP (*p* < 0.0001), ANC (*p* < 0.0018), and classical monocytes (*p* = 0.05) were significantly higher in children with obesity. CRP, ANC and classical monocytes showed positive correlations with waist circumference, insulin, HOMA-IR and triglycerides. CRP was positively associated with ANC overall (*p* = 0.05). ANC demonstrated positive correlation with monocytes (*p* < 0.001). The associations between predictor and outcome variables were influenced by sex, race, and age.

**Conclusions:** CRP and myeloid leukocyte populations, specifically classical monocytes and neutrophils associate with both body composition and metabolic parameters in children with obesity suggesting that these cells may play a critical role in metabolic impairment. Race, gender and age interactions between monocytes and metabolic parameters were significant.

## Introduction

Obesity leads to a low-grade chronic inflammation in which macrophage activation and neutrophil accumulation enhance tissue dysfunction further promoting metabolic disease (1). An example of this is insulin resistance that is exaggerated due to adipocyte dysfunction secondary to effects of macrophage activation in the adipose tissue. This leukocyte activation in adipose tissue is caused from both local signals that lead to expansion but also systemic signals that propel hematopoietic stem cell expansion and production of monocytes and neutrophils from granulocyte/macrophage progenitors (2). The net result is the sustained accumulation and activation of adipose tissue macrophages (ATMs) which shift from an anti-inflammatory, and insulin sensitive, phenotype toward a pro-inflammatory, and insulin resistant, phenotype (1, 3). These activated ATMs secrete pro-inflammatory cytokines and chemokines, that magnify recruitment of neutrophils and macrophages, myeloid cells, into the visceral adipose tissue (1, 3–5). This local inflammation subsequently contributes to an insulin resistant environment, and causes metabolic and cardiovascular complications including hyperinsulinemia, impaired glucose tolerance, hypertension, hypertriglyceridemia, and low HDL cholesterol (6). An inflammatory phenotype is also present in other tissues and may lead to metabolic dysfunction starting as early as childhood (1, 7).

Circulating monocytes are precursors of these tissue macrophages and can be divided into three populations based on their expression of CD14 and CD16: classical (CD14^++^CD16^−^), intermediate (CD14^++^CD16^+^) and non-classical (CD14^+^CD16^++^) (8). Investigations have identified that the origin of these monocytes is directly due to expansion of myeloid precursors in the bone marrow that lead to increase in both monocytes and neutrophil populations (2, 9). The activated monocyte population (typically CD14^++^ classical monocytes) is associated with cardiovascular disease in adults (10), and with increased gene expression for monocyte activation in children that have elevated markers of cardiovascular disease (11). However, it is unclear whether monocyte types are predictive of which children are at risk for metabolic disease. Several gaps also exist in translating what we know from animal and adult studies to the pediatric population. It is unclear if exposure to obesogenic diets early in life effects a specific monocyte population, or if they effect differentiation into macrophages, and if certain clinical populations are at greatest risk based on obesity class, race, gender, or age.

Surrogate inflammatory markers such as C-reactive protein and cytokines such as IL-6 have been used as predictive markers for type-2 diabetes mellitus in children with obesity (12, 13). These serum markers show that inflammation occurs in children with pre-diabetes, but do not truly identify an underlying mechanism or etiology (14). Several gaps exist in our current understanding of the mechanisms underlying pediatric obesity and metabolic disease. For example, while African American and Hispanic youth have been known to demonstrate greater insulin resistance and higher obesity rates (among other contributing factors), it is unclear if these races are at risk due to early activation of myeloid cells.

In this study, we sought to understand the association between myeloid leukocyte profiles (monocytes and neutrophils) in children and adolescents across a weight spectrum. Given prior differences in metabolic and inflammatory responses by sex, race, and age we further sought to evaluate the interaction of these factor in the relationship of inflammation with weight status.

## Materials and Methods

### Study Sample

Two hundred and eighty-second children and adolescents from ages 8 to 17 years old were recruited at the University of Michigan clinical sites, non-University of Michigan clinical sites, schools in the local area, and the University of Michigan Medical School campus. Overall, this was a convenience sampling with systematic recruitment of children with overweight and obesity. The Institutional Review Board at the University of Michigan provided ethical approval, patients provided written assent with written informed consent from patient or guardian.

Exclusion criteria included diagnoses of diabetes mellitus, acute or chronic infections, treatment with medications known to affect glucose metabolism (oral steroids, metformin, insulin or sulfonylureas), or a verbal report of being pregnant at the time of recruitment. Children and adolescents with a previous diagnosis of pre-diabetes under lifestyle therapy only (diet and exercise) or taking antihypertensive or lipid-lowering medications were not excluded. In addition, self-report and medical record review were used to confirm no current/active illness at the time of visit. Participants were allowed to participate if they had fully recovered from illness.

Based on the Centers for Disease Control and Prevention BMI-for-age growth charts (15), participants were classified as normal weight (BMI at or above the 5th and below the 85th percentile), overweight (BMI at or above the 85th and below the 95th percentile), or children with obesity (BMI at or above the 95th percentile). Participants were evaluated after a 12-h fasting state. Vital signs and anthropometrics including height, weight and waist circumference were collected. BMI percentiles for age and BMI Z-scores were calculated using the CDC SAS program (15).

Intravenous blood samples were collected in EDTA tubes and kept room temperature for flow cytometry essays. All other assays were performed using standard operating procedures from the Michigan Diabetes Research Center (MDRC) (glucose and insulin were from sodium heparin tubes placed on ice, HbA1c from EDTA tube whole blood stored at −80, lipid profiles and High sensitivity C-reactive protein (hsCRP) samples were collected in 10 ml Red top tubes kept at room temperature for 30 min before separation and freeing serum). Total cholesterol (cholesterol enzymatic end point method), HDL and LDL cholesterol (two step-direct method), triglyceride (GPO-PAP method), and glucose (hexokinase method) run on Randox RX Series Daytona chemistry analyzer. HbA1C is determined on a Tosoh G7 HPLC Analyzer (Tosoh Biosciences Inc, South San Francisco, CA). hsCRP analysis on the Siemens Advia 1,800 is performed utilizing a latex enhanced immunoturbidimetric assay. Insulin run on a double-antibody radioimmunoassay using an 125I-Human insulin tracer (Linco Research). 2-hour Oral Glucose Tolerance Test (OGTT) was performed, where glucose and insulin were measured 30, 60, 90, and 120 min after the glucose load (1.75 mg/kg up to a max of 75 g). Insulin sensitivity was estimated by the homeostasis model assessment (HOMA-IR = [fasting glucose (mg/dL)] × [fasting insulin (μU/mL)] ÷ 405).

**Figure 1:**
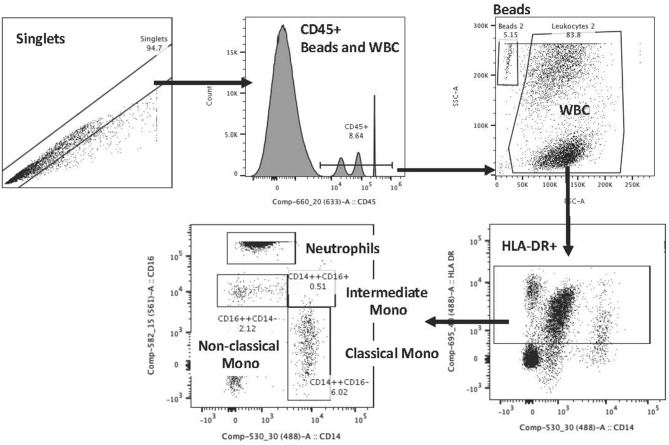
Flow cytometry gating scheme for monocyte evaluations.

Flow cytometry staining on blood samples included CD45, HLA-DR, CD14, and CD16 staining to identify neutrophils and monocyte subtypes (classical CD14^++^CD16^−^; intermediate CD14^++^CD16^+^; non-classical CD14^+^CD16^++^). Staining was done based on the Heimbeck et al. protocol (16). The antibodies that were used included CD16 PE (Beckman; IM1238U), CD14 FITC (Beckman; 6603511), CD45 APC (Beckman; IM2473U), HLA-DR PC5 (Beckman; IM2659U) with the following isotype controls: IgG1 (Mouse) PE (Beckman; IM0670U), IgG2b (Mouse) FITC (Beckman; 6603034), IgG1 (Mouse) APC (Beckman; IM2475U), IgG1 (Mouse) PC5 (Beckman; IM2663U). Staining was performed on blood samples, collected in K3 EDTA vacutainers, and were processed within 1 to 4 h of collection. Tubes were gently inverted and at room temperature antibodies were added to 50 ul of blood for unstained, isotype, single stain control and full mixture of antibodies to stain monocyte subtypes. After 20 min of staining, 100 ul of Optilyse B (Beckman; IM1400) were added to each tube for 10 min for RBC lysis and fixation and then 1000 ul of deionized water were added to each tube. 50 ul of CountBright beads (Invitrogen; C36950) were added and samples were then placed in 4°C until analyzed on a BD Aria flow cytometer. We used controls per each stain set and established consistent gaiting for experiments. Variability was reduced through the use of two individuals for staining, a single analyzer blinded to group information during the analysis and evaluation of controls with each run. The reference for the gating strategy is Heimbeck et al. (16) and Poitou et al. (17), but an example of this gating is shown in Figure 1.

### Study Variables

1. Patient weight status: normal weight, overweight, obesity (based on BMI percentile for age).
2. Demographics: sex, age, race, and ethnicity.
3. Predictor variables: Absolute neutrophil counts, absolute monocyte counts, %CD14^++^CD16^−^ monocytes (classical), %CD14^++^CD16^+^ monocytes (intermediate), %CD14^+^CD16^++^ monocytes (non-classical) and CRP.
4. Outcome variables: Waist circumference, glucose (fasting, 60 and 120 min from OGTT), insulin (fasting), HOMA-IR, HbA1c (%), fasting lipid profile (triglycerides, HDL, LDL, and total cholesterol). Fasting lipid profiles were only performed in a subset of the participants.

### Statistical Analysis

All statistical analyses were completed using *SAS software, Version 9.4, Cary, NC, USA*. Statistical outliers for monocyte counts, absolute neutrophil counts and CRP were defined as those ±3 standard deviations from the mean. 22 (1.2%) of 1,788 values were identified as outliers and were set to missing prior to analysis. We checked the distributions of predictor and outcome variables for normality prior to analysis and used non-parametric tests when necessary.

Descriptive statistics were used to describe the sample (Table 1). Pearson correlation coefficients were used to determine the relative strength of associations between predictor and outcome variables in this cross–sectional study Wilcoxon two-sample tests were used to determine if there were differences in predictor variables based on sex. To check if the associations between predictor and outcome variables were influenced by child race, we tested for the interaction between three-category child race and predictor (using SAS Proc GLM method of multiple linear regressions with interaction terms). When the interaction term was significant, we ran subgroup analysis to determine the direction and strength of effect in each child race group. We used the same method to check for the influence of child age (<12 vs. >12 years of age) on each predictor-outcome A *p*-value threshold of < 0.05 was considered significant.

**Table 1:** Demographic characteristics of the study sample.

	**All participants (*n* = 282)**	**Male (*n* = 119)**	**Female (*n* = 163)**	**>12 years (*n* = 169)**	**≤12 years (*n* = 113)**
Normal weight	47 (17%)	20 (17%)	27 (17%)	32 (19%)	15 (13%)
Overweight	73 (26%)	33 (28%)	40 (24%)	39 (23%)	34 (30%)
Obesity	162 (57%)	66 (55%)	96 (59%)	98 (58%)	64 (57%)
Age (years)	12.8 (2.5)	12.7 (2.4)	12.9 (2.6)	14.6 (1.3)	10.3 (1.4)
Height (cm)	159.2 (13.0)	161.8 (15.1)	157.3 (10.9)	166.4 (7.8)	148.4 (11.8)
Weight (kg)	74.2 (28.0)	73.8 (29.4)	74.5 (27.0)	86.6 (27.1)	55.7 (16.8)
BMI (kg/m^2^)	28.6 (8.3)	27.3 (7.7)	39.5 (8.6)	31.1 (9.0)	24.8 (5.0)
BMI percentile	90.3 (16.4)	88.3 (20.5)	91.8 (12.4)	89.9 917.1)	90.9 (15.4)
BMI Z-score	1.7 (0.8)	1.6 (0.9)	1.7 (0.7)	1.7 (0.9)	1.6 (0.8)
Waist circumference (cm)	93.4 (19.1)	92.6 (18.5)	94.0 (19.6)	100.0 (20.3)	84.0 (12.3)

*Data represent n (percentage) or mean ± standard deviation*.

## Results

Two hundred and eighty-second participants between 8 and 17 years of age were included in the final analysis (119 boys-42%, 163 girls-58%). the average age of participants was 12.8 years (Table 1) and based on the BMI percentile for age and sex, 17% of subjects had a normal weight, 26% were overweight and 57% were in the obese category. The demographic characteristics of the sample are shown in Tables 1, 2. given that some enrolled participants were unable to participate in all aspects of the study only participants with key outputs BMI, fasting glucose/insulin and inflammatory parameters were kept in the study. although several participants were missing lipids we analyzed if there were differences between the set of analysis for those with and those without lipid values and did not find significant differences in these groups and hence included all of these participants.

**Table 2A:** The mean and standard deviation (SD) of predictor and metabolic outcome variables calculated over the number of participants (N) included.

	**Female** **>** **12 years**			**Female** **≤ 12 years**		
	**Normal (*n* = 17)**	**Overweight (*n* = 20)**	**Obese (*n* = 64)**	**Normal (*n* = 10)**	**Overweight (*n* = 20)**	**Obese (*n* = 32)**
Height (cm)	162.8 (5.4)	162.8 (5.2)	163.0 (4.9)	149.4 (15.6)	144.8 (9.9)	149.7 (11.2)
Weight (kg)	59.4 (5.8)	69.3 (6.3)	98.7 (23.6)	43.3 (12.9)	46.2 (9.1)	64.6 (15.6)
BMI (kg/m^2^)	22.4 (1.5)	26.1 (1.6)	37.0 (8.2)	19.0 (2.5)	21.8 (1.5)	28.4 (3.9)
BMI percentile	73.0 (14.2)	90.4 (3.0)	98.1 (1.6)	67.8 (24.8)	90.6 (3.4)	98.1 (1.3)
BMI Z-score	0.66 (0.41)	1.3 (0.2)	2.2 (0.4)	0.44 (0.84)	1.3 (0.2)	2.2 (0.3)
Waist circumference (cm)	81.0 (5.3)	89.9 (20.2)	109.4 (18.1)	72.5 (9.8)	76.5 (7.2)	91.5 (10.4)
Absolute neutrophil count (cells/μL)	4094 (2438)	3517 (1948)	4903 (2017)	4592 (1896)	3524 (1868)	3920 (1867)
Absolute monocyte count (cells/μL)	637.4 (315)	486 (270)	671.4 (279)	871 (371.2)	620 (287)	644.7 (308.7)
%CD14^++^CD16^−^ (classic)	79.2 (9.3)	80.7 (8.7)	82.0 (7.5)	74.4 (12.0)	78.0 (10.0)	77.8 (10.1)
%CD14^+^CD16^++^ (non-classic)	14.8 (7.0)	13.4 (9.6)	13.7 (7.5)	20.5 (11.1)	16.8 (9.1)	16.9 (2.10)
%CD14^++^CD16^+^ (intermediate)	4.9 (2.9)	5.6 (3.7)	4.4 (2.8)	5.2 (2.4)	5.2 (2.9)	4.5 (2.1)
CRP (mg/L)		1.8 (2.8)^##^	5.7 (6.2)^####^		1.3 (2.4)^#^	3.1 (3.5)^###^
Fasting glucose (mg/dL)	80.6 (7.8)	80.5 (7.4)	83.5 (8.1)	79.8 (6.2)	85.8 (8.8)	83.3 (8.9)
Glucose 30 min (mg/dL)	115.4 (20.7)	111.9 (23.1)	123.1 (19.7)	129.5 (11.6)	126.6 (20.4)	124.0 (17.5)
Glucose 60 min (mg/dL)	105.1 (23.6)	98.5 (29.0)	115.6 (28)	118.4 (13.1)	115.8 (27.0)	107.6 (23.4)
Glucose 90 min (mg/dL)	104.1 (20.4)	97.9 (26.2)	110.3 (25.6)	98.7 (16.9)	106.3 (23.8)	112.3 (21.6)
Glucose 120 min (mg/dL)	102.6 (28.5)	91.9 (22.1)	106.9 (19.6)	92.5 (18.2)	108.1 (16.8)	105.3 (17.7)
Fasting insulin (μU/mL)	15.0 (5.3)	19.3 (10.1)	31.7 (19.1)	14.6 (5.5)	16.4 (6.0)	27.2 (9.4)
Insulin 30 min (μU/mL)	125.8 (89)	139.3 (98.2)	267.1 (165.8)	112.7 (40.4)	133.7 (68.0)	242.0 (111.8)
Insulin 60 min (μU/mL)	96.8 (60.5)	120.5 (82.8)	230.2 (160.7)	100.1 (59.3)	110.8 (73.9)	163.5 (115.2)
Insulin 90 min (μU/mL)	92.4 (52.1)	119.8 (100.3)	215 (192.2)	78.8 (66.2)	94.8 (53.9)	198.3 (113.8)
Insulin 120 min (μU/mL)	82.1 (61.6)	106.7 (87.8)	212.4 (157.6)	66.0 (48.4)	96.1 (46.7)	172.0 (100.9)
HbA1c (%)	5.1 (0.3)	5.1 (0.3)	5.3 (0.3)	5.1 (0.2)	5.2 (0.2)	5.2 (0.2)
HOMA-IR	3.0 (1.3)	3.9 (2.2)	6.6 (4.0)	2.9 (1.1)	3.5 (1.5)	5.6 (1.9)
Triglycerides (mg/dL)		65.5 (26.4)^**^	107.5 (53)		66.7 (35.5)^*^	95.7 (47.3)
HDL (mg/dL)		40.1 (8.7)^**^	38.3 (11.5)		39.8 (8.3)^*^	39.4 (10.3)
LDL (mg/dL)		91.4 (29.7)^**^	96.8 (26.8)		90.8 (28.4)^*^	95.5 (26.5)
Total cholesterol (mg/dL)		144.8 (25.6)^**^	147.4 (30)		140.8 (25.9)^*^	143.8 (24.4)

*Data represent mean ± standard deviation*.

# *n = 15*,

* *n = 17*,

## *n = 13*,

** *n = 18*,

### *n = 29*,

#### *n = 59*.

**Table 2B:** The mean and standard deviation (SD) of predictor and metabolic outcome variables calculated over the number of participants (N) included.

	**Male** **>** **12 years**			**Male** ** < =** **12 years**		
	**Normal (*n* = 15)**	**Overweight (*n* = 19)**	**Obese (*n* = 34)**	**Normal (*n* = 5)**	**Overweight (*n* = 14)**	**Obese (*n* = 32)**
Height (cm)	167.4 (10.1)	170.5 (7.8)	173.9 (7.4)	130.8 (6.3)	151.4 (12.1)	150.5 (10.4)
Weight (kg)	56.7 (9.9)	73.7 (7.7)	107.8 (27.5)	28.1 (4.0)	50.1 (10.5)	63.3 (15.6)
BMI (kg/m^2^)	20.2 (2.4)	25.3 (1.5)	35.4 (8.2)	16.3 (1.4)	21.6 (1.4)	27.5 (4.1)
BMI percentile	51.7 (28.7)	91.7 (3.3)	98.3 (1.6)	48.3 (27.8)	91.2 (2.8)	97.8 (1.4)
BMI Z-score	0.02 (0.8)	1.4 (0.2)	2.3 (0.5)	−0.15 (0.97)	1.4 (0.2)	2.1 (0.3)
Waist circumference (cm)	79.1 (6.1)	88.4 (6.7)	113.3 (17.2)	63.6 (5.7)	78.7 (5.8)	89.9 (10.4)
Absolute neutrophil count (cells/μL)	3890 (2421)	3761.1 (1846)	4016 (2051)	3365 (1207)	2485 (1059)	4512 (2257.6)
Absolute monocyte count (cells/μL)	760.3 (310.3)	724.1 (417.4)	564.4 (201.4)	664 (281.6)	556 (230.5)	750.5 (325.2)
%CD14^++^CD16^−^ (classic)	82.0 (7.3)	75.7 (11.5)	81.7 (8.3)	78.7 (8.2)	76.2 (9.4)	79.5 (10.3)
%CD14^+^CD16^++^ (non-classic)	14.3 (6.9)	17.1 (8.4)	14.3 (7.9)	17.5 (7.2)	16.7 (7.5)	14.8 (8.3)
%CD14^++^CD16^+^ (intermediate)	3.6 (2.1)	5.2 (3.2)	4.0 (1.9)	3.7 (1.2)	5.2 (2.2)	4.6 (2.3)
CRP (mg/L)		1.4 (1.7)^##^	2.8 (3.2)		0.27 (0.25)^#^	2.3 (2.3)
Fasting glucose (mg/dL)	84.9 (6.2)	83.7 (7.6)	87.6 (8.4)	85.4 (7.7)	81.6 (6.8)	82.2 (6.7)
Glucose 30 min (mg/dL)	132.1 (22.3)	126.7 (18.2)	129.5 (24.1)	151.4 (42.5)	118.4 (23.6)	129.5 (20.8)
Glucose 60 min (mg/dL)	113.1 (27.2)	113.8 (23.9)	120.2 (32.8)	114.8 (27.4)	97.1 (20.3)	111.7 (23.9)
Glucose 90 min (mg/dL)	99.1 (15.2)	103.8 (22.4)	111 (31.4)	97.8 (22.2)	97.2 (21.5)	104.4 (19.4)
Glucose 120 min (mg/dL)	92.2 (15.0)	94.0 (20.5)	103.7 (30.9)	91.4 (23.2)	99.0 (17.5)	104.3 (15.0)
Fasting insulin (μU/mL)	15.3 (5.0)	16.3 (4.5)	31.4 (15.1)	24.6 (37.2)	22.4 (26.7)	22.8 (10.6)
Insulin 30 min (μU/mL)	139.2 (76.1)	115.5 (46.1)	277.6 (137.7)	99.9 (69.4)	113.3 (52.0)	181.1 (134.8)
Insulin 60 min (μU/mL)	102.2 (36.2)	98.2 (38.5)	241.4 (139)	55.9 (34.2)	68.2 (38.3)	132.5 (105.8)
Insulin 90 min (μU/mL)	77.8 (24.0)	81.8 (37.1)	177.8 (120.6)	41.8 (39.2)	65.1 (38.8)	120.7 (103)
Insulin 120 min (μU/mL)	68.7 (30.8)	65.9 (37.0)	143.9 (130.3)	35.1 (26.7)	66.3 (41.0)	115.9 (87.4)
HbA1c (%)	5.1 (0.3)	5.0 (0.2)	5.3 (0.4)	5.1 (0.2)	5.2 (0.3)	5.3 (0.3)
HOMA-IR	3.2 (1.0)	3.4 (1.1)	6.7 (3.2)	5.7 (9.1)	4.6 (5.7)	4.6 (2.3)
Triglycerides (mg/dL)		76.5 (44.2)^*^	114.2 (56.9)		61.8 (31.3)	82.3 (45.9)
HDL (mg/dL)		38.4 (8.1)^*^	35.1 (7.4)		42.1 (12.5)	37.5 (7.4)
LDL (mg/dL)		92.9 (18.5)^*^	97.5 (28.2)		96.5 (23.4)	99 (24.9)
Total cholesterol (mg/dL)		137.1 (18.7)^*^	145.1 (32.4)		146.1 (29.2)	144.3 (21.4)

*Data represent mean ± standard deviation*.

# *n = 13*,

## *n = 16*,

* *n = 17*.

### Relationship Between Predictor (Monocytes, Neutrophils and CRP) and Metabolic Outcome Variables

All correlations between predictor and outcome variables in all participants are shown in Table 3 with average and standard deviation (SD) shown in Table 2 by sex, age and weight status. There was a significant positive correlation with BMI and BMI Z-score alone with waist circumference, fasting insulin, HbA1c, HOMA-IR and triglycerides but a negative correlation with HDL cholesterol. CRP showed a positive correlation with waist circumference, fasting insulin, HbA1c, HOMA-IR, and triglycerides (Table 3), consistent with prior studies demonstrating an increased CRP with weight status (*p* < 0.0001) (18, 19).

**Table 3:** Correlation coefficients (R) between predictor and metabolic outcome variables.

	**Neutrophils**	**%CD14^**++**^CD16^**−**^ (classical)**	**%CD14^**+**^ CD16^**++**^ (non-class.)**	**%CD14^**++**^CD16^**+**^ (Inter.)**	**All monocytes**	**CD14^**++**^CD16^**−**^ (classical) Cells/ul**	**CD14^**+**^CD16^**++**^ (non-class.) Cells/ul**	**CD14^**++**^CD16^**+**^ (Inter.) Cells/ul**	**CRP**
Waist circumference	^**^0.18	^**^0.19	^**^-0.16	−0.04	−0.02	−0.01	^*^-0.13	−0.11	^***^0.33
Fasting glucose	0.09	^*^0.12	−0.08	−0.04	−0.03	−0.09	−0.09	−0.10	0.13
Fasting insulin	^**^0.21	^*^0.14	^*^-0.14	−0.00	0.11	0.04	−0.06	−0.03	^*^0.17
HbA1c	0.05	−0.02	0.09	−0.1	−0.01	−0.08	0.009	^*^-0.12	^***^0.26
HOMA-IR	^***^0.22	^**^0.16	^*^-0.15	−0.00	0.09	0.05	−0.07	−0.04	^**^0.19
Triglycerides	^*^0.17	^***^0.24	^***^-0.24	−0.05	0.03	0.04	^*^-0.16	−0.04	^*^0.15
HDL	−0.10	−0.12	0.07	^*^0.15	^*^-0.17	−0.09	−0.04	0.13	−0.12
LDL	−0.01	0.04	−0.05	^*^-0.14	0.12	0.02	0.05	−0.05	−0.12
Total cholesterol	−0.05	0.03	−0.04	−0.06	−0.04	−0.04	−0.05	−0.04	−0.00

* *p < 0.05*,

** p < 0.01

*** *p < 0.001*.

*Bold values represent all correlations that were statistically significant*.

Absolute neutrophil count (ANC) was significantly higher in children with obesity (*p* = 0.002) compared to non-obese participants, consistent with what has been seen in other clinical studies (18, 19) demonstrating validity of this finding in our study. There was a positive correlation between ANC and waist circumference, fasting insulin, HOMA-IR, and triglycerides (Table 3). Neutrophils did not associate with glucose levels (fasting, during glucose tolerance test or as HbA1c) or the rest of the lipid profile.

**Figure 2:**
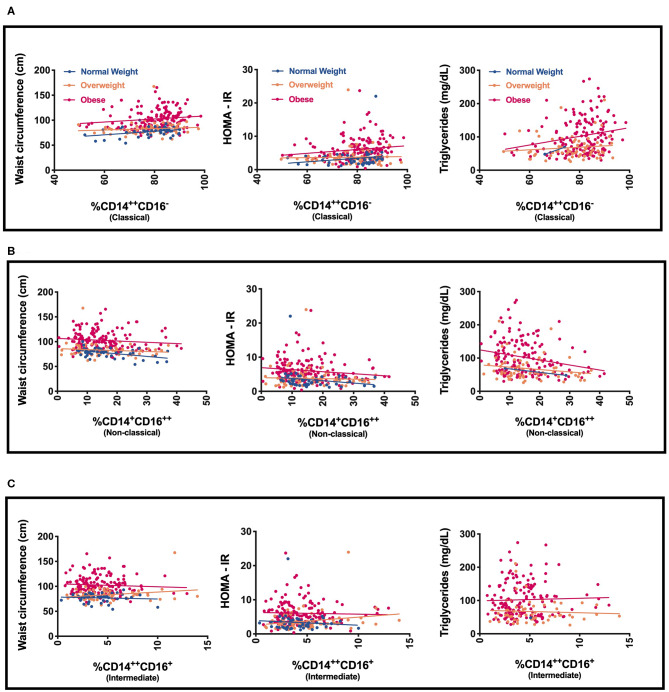
Relationship of monocyte populations with waist circumference, HOMA-IR, and triglycerides **(A)** % CD14^++^CD16^−^ (Classical monocytes), **(B)** % CD14^+^CD16^++^ (Non-classical monocytes), and **(C)** % CD14^+^CD16^++^ (Non-classical monocytes).

**Figure 3:**
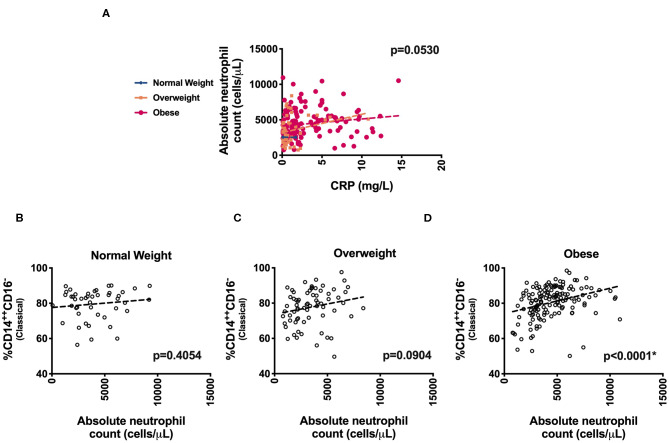
Association of inflammatory markers. Distribution of **(A)** absolute neutrophil counts (ANC) and CRP levels and **(B–D)** % CD14^++^CD16^−^ (Classical monocytes) with ANC across weight categories.

We next evaluated specific monocyte populations. Classical monocytes (*p* = 0.05) were significantly higher in children with obesity. We found a positive correlation between %CD14^++^CD16^−^ (classical) monocytes and waist circumference, fasting glucose, fasting insulin, HOMA-IR, and triglycerides (Table 3 and Figure 2). There was a negative correlation between %CD14^++^CD16^+^ (intermediate) monocytes and LDL and a positive association with HDL (Table 3). There was also a negative correlation between %CD14^+^CD16^++^ (non-classical) monocytes and waist circumference, fasting insulin, HOMA-IR, and triglycerides (Table 3, Figure 2). To understand which inflammatory factors are changed together, we evaluated the associations of ANC and CRP with the different monocyte populations and identified that CRP trended with ANC overall (*p* = 0.05) (Figure 3). ANC was significantly associated with monocytes overall consistent with an upregulation in these myeloid cells together (*p* < 0.001). The relationship between ANC and % classical monocytes was only significant in children with obesity but trended toward an association in children affected by overweight (Figure 3). Overall, these results demonstrate that classical monocytes and neutrophils are increased with weight status in children, however, we are unable to determine causality in this association.

### Differences by Sex

No difference in BMI Z-score (*p* = 0.57), CRP (*p* = 0.11), absolute neutrophil count (*p* = 0.10), absolute monocyte count (*p* = 0.46), or percent of monocytes in each category were seen when compared by sex. However, we did see a stronger association of waist circumference and ANC in females (*p* = 0.0159).

### Differences by Race

**Figure 4:**
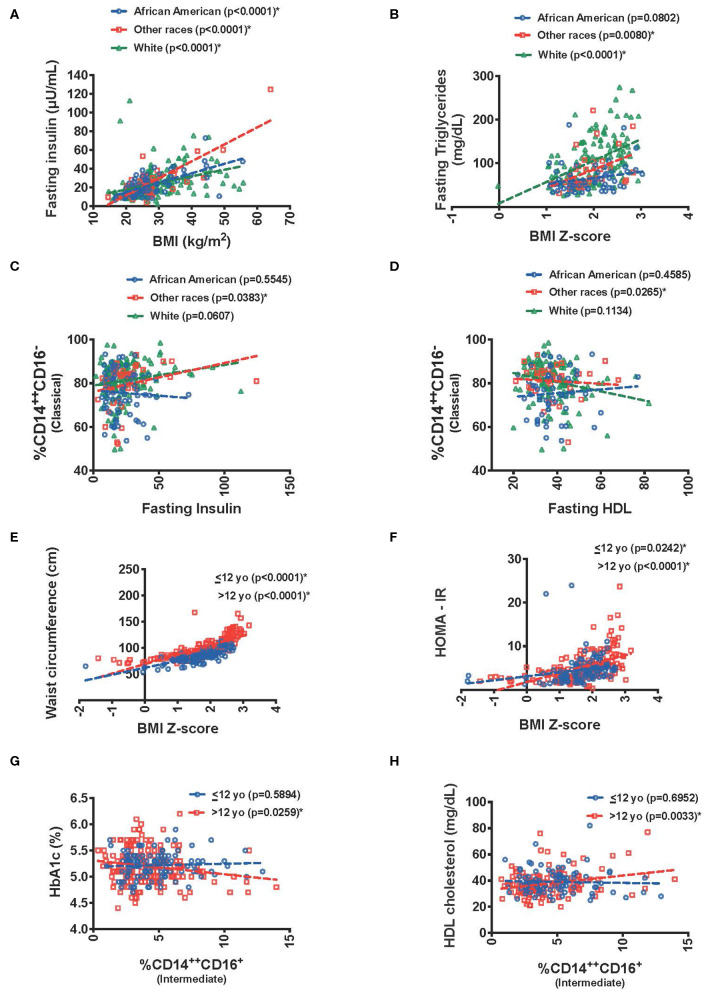
Graphical display of effect of race/ethnicity and age interactions between predictor and metabolic outcome variables. Relationship between **(A)** fasting insulin and BMI, **(B)** fasting triglycerides and BMI Z-score, **(C)** classical monocytes and fasting insulin, **(D)** classical monocytes and fasting HD with race/ethnicity interactions. Relationship between **(E)** waist circumference with BMI Z-score, **(F)** HOMA-IR with BMI Z-score, **(G)** HbA1C(%) with intermediate monocytes, and **(H)** fasting HDL with intermediate monocytes with age interactions. *The “Other race” category includes: All participants who reported Asian and Hispanic race, and who reported more than one race.

Given the paucity of studies focusing on race/ethnicity an exploratory analysis was undertaken with our data. We used a three-category race grouping of White (56 %), African-American (24%) and other races (17 %) [included all participants who reported Asian (3%) or Hispanic race (7%), and all who reported more than one race (7%)]. There was a significant interaction between race and BMI (kg/m2) in predicting fasting insulin (*p* = 0.0033). There was an association between higher BMI and higher fasting insulin in African Americans (*p* ≤ 0.0001), whites (*p* ≤ 0.0001), but for other races the slope was steeper (*p* ≤ 0.0001) (Figure 4A). Similar results were seen with BMI and HOMA-IR (0.0344). Interestingly, BMI-Z score was associated with higher fasting triglycerides in the White race category (*p* = 0.0255) (Figure 4B).

When evaluating insulin resistance there was a significant interaction in the association of fasting insulin with % CD14^++^CD16^−^ (classical) monocytes (*p* = 0.0365) and HOMA-IR (*p* = 0.0403), due to a significant association in the other race category (*p* = 0.0383 and 0.0277, respectively) (Figure 4C). The other race category also drove the % CD14^++^CD16^−^ (classical) monocytes association with lower HDL (*p* = 0.0161 for the interaction and *p* = 0.0265 for the other race association) (Figure 4D).

### Differences by Age

Given that puberty is associated with changes in growth and sex hormones that possibly influence inflammatory responses during obesity, we evaluated our study group by age. Although we were unable to evaluate puberty status in this cohort, we evaluated the influence of age (<12 vs. >12 years of age) on each predictor-outcome association and found the following interactions that were significant predictors in the overall analysis. We chose this cut-off to try to identify an age cut-off where most children would have initiated puberty (20–22).

As a measure to how the data associated those >12 years of age had higher waist circumference for BMI-Z score then those <12 (*p* = 0.0096). Interestingly, BMI z-score also had a stronger association to fasting insulin, HOMA-IR, and fasting total cholesterol in those > 12 years of age (*p* = 0.0225, *p* = 0.0134, and *p* = 0.0237, respectively) (Figures 4E,F).

When evaluating this interaction by age group, there was a trend toward a significant interaction between %CD14++CD16+ intermediate monocytes and HbA1c (*p* = 0.0528) (Figure 4G) with a negative association in those >12 years old and a higher HDL cholesterol in those >12 years old with elevated intermediate monocytes (*p* = 0.0244) (Figure 4H).

## Discussion

Obesity and its medical sequelae starting in pediatric ages are significant concerns making biomarkers critical for future metabolic disease prediction. The quantitative results in this study emphasize that classical monocytes, absolute neutrophil count, and C-reactive protein are associated with metabolic impairment and cardiovascular risk factors. The novelty of this study is that it identifies differences of monocyte subtypes in obesity induced metabolic derangement by sex, race and age in children and adolescents. This study also furthers our understanding by demonstrating that the ANC increase is specifically associated with changes in inflammatory classical monocytes, emphasizing that even in children, there may be global changes in myelopoiesis with obesity. This lays the groundwork for future studies focused on understanding the pathophysiologic mechanisms that drive myelopoiesis with obesity and identify which children are at greater risk for inflammatory responses.

Multiple inflammatory markers have been implicated in playing a role in linking obesity to metabolic impairment in children and adolescents (18, 19, 23–25). We examined the relationship of CRP and myeloid leukocyte cells on the metabolic derangement caused by childhood obesity. We found that CRP and ANC were positively associated waist circumference, insulin, HOMA-IR and triglycerides. We uniquely also examined the roles of the monocytes on childhood obesity. In adult studies, activated CD14^++^ monocytes are associated to obesity and its metabolic and cardiovascular complications (11, 17, 26, 27). This classical monocyte population is thought to reflect the pro-inflammatory macrophages seen in metabolic tissues with obesity (28). Although CD14^++^CD16^−^ (classical) monocytes were detectable in children with obesity in prior studies (11, 29), the link between monocytes and metabolic impairment has not been clear. In our study, we showed a positive link between CD14^++^CD16^−^ (classical) monocytes and waist circumference, metabolic parameters of insulin, HOMA-IR and triglycerides. Based on these results, we conclude that the proportion of classical monocytes in circulation might be used for early detection and prevention of these metabolic complications and should be further profiled to understand how they are changing qualitatively with obesity as this may directly link to the mechanisms of tissue dysfunction with obesity.

The role of intermediate monocytes in obesity remains unclear although examined in obese adults and associated with cardiovascular disease (15, 17). According to previous limited studies, there are increasing numbers of intermediate monocytes in childhood obesity (11, 29). We were not able to see a significant association of this population with metabolic measures, but this may be due to differences in this study population or the fasting nature of the blood collection. In adult men this intermediate monocyte CD14^++^CD16^+^ population can decrease with an energy restriction diet (30). Further a reduction of fat mass is associated with a decrease of CD14^dim^CD16^+^ (non-classical) and CD14^+^CD16^+^ subsets (17). This decrease of the CD14^+^CD16^+^ monocytes noted during weight loss was associated with a decrease in the intima-media thickness and hence a reduced risk in atherosclerosis. The role of non-classical monocytes also remains unclear in children with obesity (11, 29). In our study, there is a resulting negative association with the non-classical monocytes and fasting insulin and lipids. To our knowledge, this is the first study suggesting that a shift from intermediate and non-classical monocytes toward classical monocytes may lead to impaired metabolic health and a shift to these non-classical and intermediate types may actually be protective.

The link between different white blood cell populations is a unique area of our study. The relationship between classical monocytes with ANC in a stepwise progression in BMI until obesity (Figure 3) demonstrates that these two cell types are increasing together just as we have seen in animal studies (2) further demonstrating a link in alterations in how immune cells are developed with dietary obesity. However, our data here suggest that these findings may be affected by age and race. In our study, we explored the association between metabolic dysfunction and obesity was greater in those older than 12 years of age (Figure 4). These data suggest that pathophysiologic mechanisms related to the obesity induced metabolic impairment are already operative in childhood and progress from insulin resistance to impaired glucose and lipid metabolisms as children get older. With the lack of appropriate pubertal staging, we were only able to evaluate age using a chosen cut-off of 12 based on what has been published for when puberty has occurred in lean and obese individuals (31–33). There are limitations in doing this given that puberty occurs differently in obesity and in both sexes.

Sex differences are also a critical piece to our findings. Although multiple studies have shown higher CRP levels in females compared to males (34–36), there are limited studies about sex differences in children with obesity (25, 37). In our study, we saw only mild differences in inflammation but did see that girls had a stronger association of waist circumference with ANC suggesting that those girls with truncal adiposity are at highest risk for inflammation as has been seen in other studies (25, 37). Overall these findings emphasize that many factors including hormones, body composition, and maturity of the immune system change with age and sex, all of which may play a role in the differences we identified which emphasize that further investigation is necessary to delineate the age and sex differences in inflammatory responses to obesity.

In addition to evaluating sex and age we performed exploratory analysis of our data to understand the impact of race on myeloid inflammatory responses. Racial and ethnic differences in the prevalence of metabolic syndrome exist, with the highest rates among Hispanics, and African Americans (35, 38). Our study demonstrated a significant interaction between BMI and fasting insulin in all races, with the steepest slope amongst other races including Hispanics. A limitation of our study was that due to small numbers in the “other races” category, this also includes Asians and those who reported more than one race. We also examined whether racial disparity in lipid profiles exist in children and adolescents. Dhuper et al. reported that the lowest HDL levels were seen in white children and adolescents however the highest HDL levels were determined in African-Americans across all BMI categories (39). In our study, we found a strong negative association between BMI and HDL in other races and a near significance in Whites, but no association in African-Americans. African Americans were also different in terms of inflammatory responses with our primary classical monocyte population (CD14^++^CD16^−^) not associating with fasting insulin as we saw in other races and whites. These differences reveal the need to identify how metabolic derangement occurs in certain racial groups with obesity in children and adolescents and that inflammatory responses may differ by race/ethnicity.

There are several limitations in the current study including issues of design and limitations in conclusions that could be made. While we excluded children with acute and chronic infections, children who were in an incubation period from their illnesses may still have altered myeloid profiles and would have been included. We also acknowledge that only fasting blood samples were collected and fed samples may have very different results, this will be a critical future study to understand if substrate differences during a meal alter inflammatory responses. The lack of samples from participants with type 2 diabetes also limits the ability to predict monocyte profiles in those already suffering from consequences of metabolic syndrome who might be at highest risk for inflammatory changes. Another limitation in study design is the lack of determination of pubertal status by clinical parameters and measures of total estrogen and free testosterone concentrations. Pubertal age may affect cardiovascular risk factors and metabolic derangement, and the variations seen in BMI-Z score and monocyte profiles due to age differences might be caused by either sex hormones or number of years of obesity. Though this is one of the largest studies of this type (29, 40–42) The single site small size is a limitation of our study. This led to us being underpowered to evaluate age as a continuous factor and limited our analysis by race. A larger sample size study is needed to elicit differences by races and have expanded information about Hispanics, Asians and participants who reported more than one race in one category. Even though our study revealed associations between myeloid leukocyte profiles and components of metabolic impairment, causality could not be investigated in this cross-sectional study.

While flow cytometry staining was used in this study and demonstrated heterogeneity in these myeloid populations this only characterizes surface markers which alone may not be best for these studies as characteristics of these cells may better associate with disease state. Further *in vitro* studies are needed to show how these monocytes cells differentiate into macrophages or further characterization of these monocytes by gene expression to validate other studies done in adults. Devevre et al. identified that classical monocytes are the monocyte population most responsive to Toll-like receptor signals and upregulate chemokines to enhance migration of these monocytes in individuals with obesity (27). Since the classical monocytes have upregulated chemokines such as CCR2 and CCR5 it is likely that this contributes to more activated tissue macrophages in the children with obesity in this cohort, and why these same children with obesity have higher insulin levels and HOMA-IR. Future studies are needed to determine the specific gene expression, inflammatory pathway activation and characteristics of the heterogeneity of monocyte populations in childhood obesity.

In conclusion, chronic low-grade inflammation; CRP, myeloid leukocyte populations, specifically classical monocytes and neutrophils associated with obesity in children and adolescents and can cause metabolic dysfunction and cardiovascular disease even in childhood. Markers of immune system activation in obesity induced chronic inflammation can be used to predict and prevent risk of metabolic and cardiovascular diseases. More studies are needed to reveal the predictive value of monocyte profiles, and to determine differences in these inflammatory markers by age, race and sex. These findings emphasize that further mechanistic studies are needed to understand how leukocytes are activated with obesity and how they can be used to improve screening, prevention, and treatment of metabolic disease during childhood and adolescence.

## Data Availability Statement

The datasets generated for this study are available on request to the corresponding author.

## Ethics Statement

The studies involving human participants were reviewed and approved by University of Michigan Institutional Review Board. Written informed consent to participate in this study was provided by the participants' legal guardian/next of kin.

## Author Contributions

KS participated in initial study design, experiment planning, conducting experiments, data analysis and interpretation, manuscript review and preparation. AB assisted in manuscript revision, data analysis and data interpretation. CL participated in initial study design, experiment planning, data analysis and interpretation, manuscript review. CG-S wrote the initial manuscript text, recruited participants and participated in review of final manuscript. EH, SW, and JL assisted in study design, participant recruitment and review of final manuscript. PW, SA, CG, AT, and TH participated in data collection and manuscript preparation. JS participated in data analysis.

## Conflict of Interest

The authors declare that the research was conducted in the absence of any commercial or financial relationships that could be construed as a potential conflict of interest.
